# The Sphingosine Kinase 1 Inhibitor, PF543, Mitigates Pulmonary Fibrosis by Reducing Lung Epithelial Cell mtDNA Damage and Recruitment of Fibrogenic Monocytes

**DOI:** 10.3390/ijms21165595

**Published:** 2020-08-05

**Authors:** Paul Cheresh, Seok-Jo Kim, Long Shuang Huang, Satoshi Watanabe, Nikita Joshi, Kinola J.N. Williams, Monica Chi, Ziyan Lu, Anantha Harijith, Anjana Yeldandi, Anna P. Lam, Cara Gottardi, Alexander V. Misharin, G.R. Scott Budinger, Viswanathan Natarajan, David W. Kamp

**Affiliations:** 1 Department of Medicine, Division of Pulmonary & Critical Care Medicine, Jesse Brown VA Medical Center, Chicago, IL 60611, USA; p-cheresh@northwestern.edu (P.C.); seokjo.kim@northwestern.edu (S.-J.K.); ziyan@northwestern.edu (Z.L.); s-buding@northwestern.edu (G.R.S.B.); 2 Department of Medicine, Feinberg School of Medicine, Northwestern University, Chicago, IL 60611, USA; swatanabe111452@gmail.com (S.W.); joshi.nikita@gmail.com (N.J.); kinolawilliams@gmail.com (K.J.N.W.); Mon.chi930@gmail.com (M.C.); Anna_Lam@vrtx.com (A.P.L.); c-gottardi@northwestern.edu (C.G.); a-misharin@northwestern.edu (A.V.M.); 3 Department of Pharmacology and Regenerative Medicine, University of Illinois at Chicago, Chicago, IL 60612 USA; huangcampus@gmail.com (L.S.H.); harijith@gmail.com (A.H.); visnatar@uic.edu (V.N.); 4 Department of Pathology, Feinberg School of Medicine, Northwestern University, Chicago, IL 60611, USA; a-yeldandi@northwestern.edu; 5 Department of Medicine, University of Illinois at Chicago, Chicago, IL 60612, USA

**Keywords:** SPHK1, mtDNA damage, oxidative stress, alveolar epithelial cell, monocytes, pulmonary fibrosis

## Abstract

Idiopathic pulmonary fibrosis (IPF) is a chronic disease for which novel approaches are urgently required. We reported increased sphingosine kinase 1 (SPHK1) in IPF lungs and that SPHK1 inhibition using genetic and pharmacologic approaches reduces murine bleomycin-induced pulmonary fibrosis. We determined whether PF543, a specific SPHK1 inhibitor post bleomycin or asbestos challenge mitigates lung fibrosis by reducing mitochondrial (mt) DNA damage and pro-fibrotic monocyte recruitment—both are implicated in the pathobiology of pulmonary fibrosis. Bleomycin (1.5 U/kg), crocidolite asbestos (100 µg/50 µL) or controls was intratracheally instilled in Wild-Type (*C57Bl6)* mice. PF543 (1 mg/kg) or vehicle was intraperitoneally injected once every two days from day 7−21 following bleomycin and day 14−21 or day 30−60 following asbestos. PF543 reduced bleomycin- and asbestos-induced pulmonary fibrosis at both time points as well as lung expression of profibrotic markers, lung mtDNA damage, and fibrogenic monocyte recruitment. In contrast to human lung fibroblasts, asbestos augmented lung epithelial cell (MLE) mtDNA damage and PF543 was protective. Post-exposure PF543 mitigates pulmonary fibrosis in part by reducing lung epithelial cell mtDNA damage and monocyte recruitment. We reason that SPHK1 signaling may be an innovative therapeutic target for managing patients with IPF and other forms of lung fibrosis.

## 1. Introduction

Idiopathic pulmonary fibrosis (IPF) is a chronic, progressive age-related lung disease that affects nearly 120,000 people in the USA and has a median survival of only 3–5 years [1,2]. Two FDA-approved drugs (pirfenidone and nintedanib) diminish the reduction in pulmonary function in patients with IPF but neither agent restores or augments lung function. Thus, there is a crucial unmet need for identifying innovative cellular and molecular pathways that can mitigate pulmonary fibrosis. The pathogenesis of pulmonary fibrosis is incompletely understood but accumulating evidence firmly implicate three important events including: (i) increased levels of bioactive lipid mediators originating from sphingosine kinase 1 (SPHK1) [1,3,4,5]; (ii) alveolar epithelial cell (AEC) apoptosis resulting from mitochondrial dysfunction and the production of reactive oxygen species (ROS) that can promote mitochondrial DNA (mtDNA) damage [1,6,7,8,9,10,11]; and (iii) recruitment of the profibrotic monocyte-derived alveolar macrophages (Mo-AMs) that are causally implicated in mediating both bleomycin- and asbestos-induced murine lung [12,13,14].

Activation of bioactive lipid mediators, such as sphingosine-1-phosphate (S1P) and their G-protein coupled receptors (S1PR1-5) are important in the pathophysiology of IPF and experimental lung fibrosis [3,4,5,15]. In mammalian cells, S1P is primarily generated by phosphorylation of sphingosine catalyzed by sphingosine kinase (SPHK) 1 and 2 [16,17,18], and degraded by S1P lyase (S1PL) and lipid phosphate phosphatases [17]. S1P metabolic enzymes, especially SPHK1 and S1PL, are ubiquitously expressed in all cells and have crucial pathogenic roles in animal models of lung fibrosis and several human diseases, including IPF [3,4,5,15,19,20]. Our group showed that S1P and S1PL are highly expressed in the lung tissues of patients with IPF and bleomycin-challenged mice and, notably, demonstrated that SPHK1 levels negatively correlated with lung function and IPF survival [3,15,20]. Furthermore, bleomycin-induced pulmonary fibrosis is mitigated by a global SPHK inhibitor or genetic deletion of *Sphk1^-/-^*, but not *Sphk2^-/-^* in mice [15]. More recently, we reported that bleomycin-induced pulmonary fibrosis is reduced by a nonspecific SPHK inhibitor (SKI-II) [15] or conditional deletion of *Sphk1* in murine AEC or fibroblasts, but not in endothelial cells [21]. SPHK1/S1P signaling activates the Hippo/Yes-associated protein (YAP) pathway and mitochondrial reactive oxygen species (ROS) production in pro-fibrotic lung fibroblasts [21]. However, it is unknown whether administration of SPHK1 specific inhibitor PF543, following exposure to fibrogenic agents can still mitigate pulmonary fibrosis and, if so, whether the SPHK1/S1P pathway impacts AEC mtDNA integrity and recruitment of pro-fibrotic Mo-AMs.

The mitochondria are the ATP-generating powerhouse of cells but are also crucial for regulating complex intracellular signals that determine whether cells live or die. Mitochondrial dysfunction and mtDNA damage can activate intrinsic AEC apoptosis in patients with IPF and appear crucial in the pathobiology of murine models of lung fibrosis, such as bleomycin (resolving model of lung fibrosis) and asbestos fibers (nonresolving model of lung fibrosis (asbestosis) [1,6,7,8,9,10,22,23,24,25,26,27,28]. We showed that mitochondrial 8-oxoguanine DNA glycosylase 1 (mtOGG1), a key mtDNA base excision repair (BER) enzyme, prevents oxidant-induced AEC apoptosis and lung fibrosis by mitigating mtDNA damage [8,29,30,31]. Furthermore, mitochondrial catalase enforced-expression (*MCAT*) transgenic mice have reduced asbestos- and bleomycin-induced lung fibrosis due in part to reductions in AT2 cell mitochondrial ROS production, mtDNA damage and apoptosis [6]. MtDNA release, which can occur from transforming growth factor-beta (TGFβ)-exposed normal human lung fibroblasts (HLF) or apoptotic AECs, is directly linked to IPF mortality [32,33]. Collectively, these data suggest that AEC mtDNA damage promotes apoptosis and lung fibrosis; however, the relationship between AEC mtDNA damage and S1P/SPHK1 signaling in the pathobiology of lung fibrosis is unclear.

We reasoned that SPHK1 inhibition by PF543 administered following exposure to a fibrogenic agent will afford substantial protection against lung fibrosis by reducing AEC mtDNA damage and recruitment of Mo-AMs. In this study, we report that PF543 reduces both bleomycin- and asbestos-induced pulmonary fibrosis when administered 7- or 14-days post challenge, and that the protective effects occur in association with diminished lung expression of profibrotic markers, lung mtDNA damage and recruitment of fibrogenic monocytes. Furthermore, unlike HLF that are resistant to asbestos-induced mtDNA damage, asbestos augments lung epithelial cell mtDNA damage and PF543 is protective. Taken together, these findings suggest that the SPHK1 signaling axis is an innovative therapeutic target for managing patients with IPF and other forms of lung fibrosis by ameliorating AEC mtDNA damage and Mo-AM recruitment.

## 2. Results

### 2.1. PF543 Administered Following Exposure to Either Bleomycin or Asbestos Mitigates Pulmonary Fibrosis

**Figure 1:**
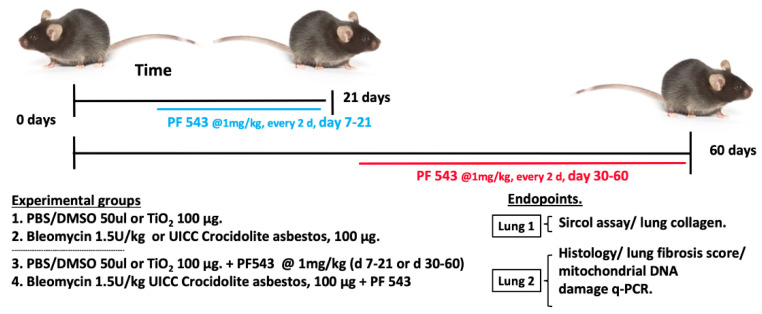
Experimental Design. For the 21-d exposure, mice were treated with a single IT instillation of either bleomycin (1.5 U/kg), crocidolite asbestos (100 µg in 50 µL), or control (bleomycin control: PBS/DMSO−50 µL; asbestos control: TiO_2_ (100 µg/ 50 µL). On day 7 after IT instillation, the mice in each group were treated with interperitoneal (IP) instillation every other day with either PF543 (1 mg/kg) or saline until day 21. For the 60-d exposure, mice were treated with either asbestos or TiO_2_ as above and on day 30, the mice in each group were treated with either IP instillation of either PF543 (1 mg/kg) or saline every other day until day 60. At the end of treatment periods, the lungs were harvested for the various listed experimental endpoints.

**Figure 2:**
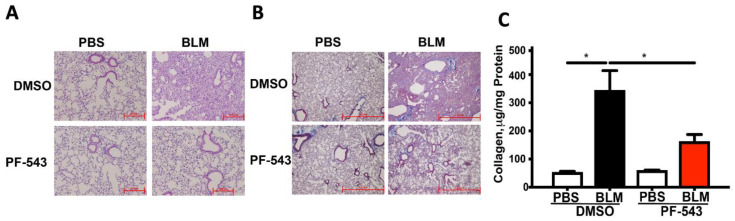
Sphingosine Kinase 1 (SphK1) inhibition post bleomycin challenge mitigates pulmonary fibrosis in mice at 21 days. Fibrosis determined on day 21 in mouse lung. Hematoxylin and Eosin (**A**) and Trichrome staining (**B**) of lung specimens. Collagen levels by Sircol assay (**C**). All *n* = 5−8; * *p* < 0.05 vs. PBS+DMSO. Scale bars: 100× = 0.2 mm (Panel A), 50× = 1.0 mm (Panel B).

**Figure 3:**
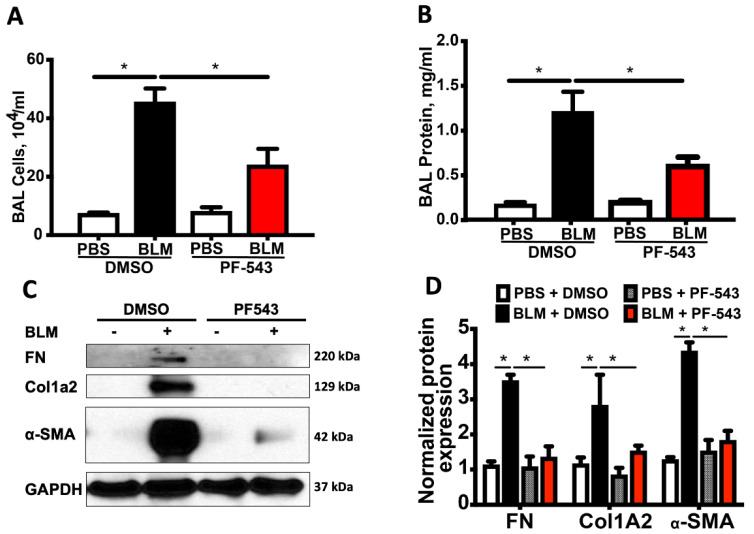
SphK1 Inhibition post challenge mitigates bronchoalveolar lavage (BAL) cells counts and protein markers of fibrosis in mice at 21 days. Fibrosis determined on day 21 (**A**) BAL cell counts. *n* = 6−8 (**B**) BAL protein and quantification of expression of fibronectin (FN), α-SMA, and collagen in lung tissues. *n* = 5−7 (**C***,***D**). All *n* = 4; * *p* < 0.05 vs. PBS+DMSO, PBS+DMSO+PF543, or PBS+ BLM+ PF543.

We previously showed that concurrent treatment of mice with a nonspecific SPHK1 inhibitor, SK1-II, at the time of bleomycin exposure reduces lung fibrosis in a manner similar to that seen in the *Sphk1^-/-^* mice [15,21]. To determine whether post-exposure to PF543, a SPHK1 specific inhibitor can reduce lung fibrosis in vivo, we used two murine pulmonary fibrosis models resulting from exposure to either bleomycin (spontaneously resolves) or amphibole crocidolite asbestos (nonresolving) [34]. A schematic model of our experimental approach is illustrated in Figure 1. As expected, a single IT-instillation of bleomycin (1.5 U/kg in 50 µL PBS) induces pulmonary fibrosis at 21 d in B6 mice as assessed by histology and lung collagen levels (Figure 2). Notably, mice treated with PF543 beginning on 7d following bleomycin exposure through 21 d had significantly reduced lung fibrosis at 21 d (Figure 2) that was accompanied by significant reductions total cells in BAL fluid and protein levels (Figure 3A,B) compared to PF543 untreated animals. Furthermore, PF543 post-exposure treatment reduced lung tissue expression of profibrotic markers including fibronectin, collagen-1A2 (Col1A2), and smooth muscle actin(α-SMA) (Figure 3C,D) please see Figure S2 for uncropped western blots.

**Figure 4:**
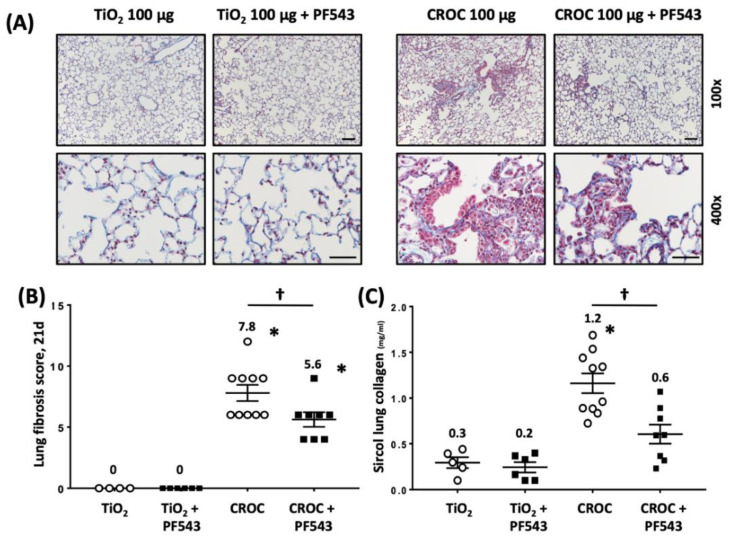
Sphk1 inhibition 7-days post-asbestos challenge attenuates pulmonary fibrosis in mice at 21 days. Fibrosis determined on day 21 in mouse lung. Trichrome staining (**A**) and fibrosis score (**B**) of lung specimens. Collagen levels by Sircol assay (**C**). *n* = 4−11; * *p* < 0.05 vs. Ti or Ti+PF543; ^†^ *p* < 0.05 vs. Crocidolite asbestos (CROC). Scale bars: 100× = 0.1mm; 400× = 0.05 mm.

**Figure 5:**
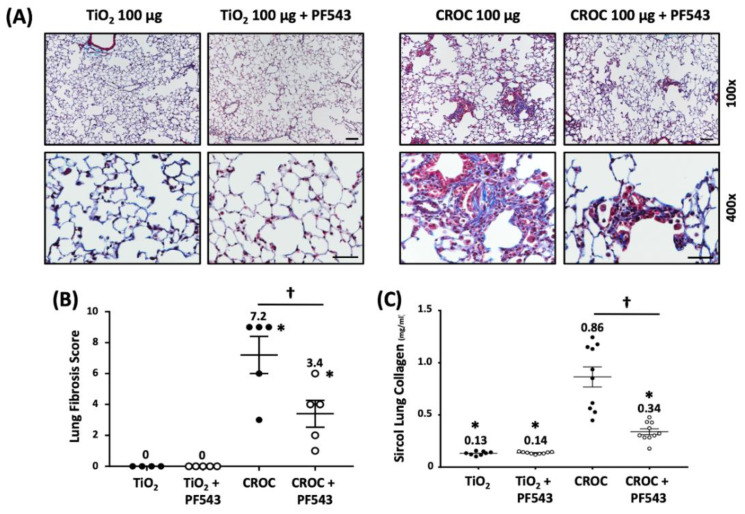
PF543 given at 30-days post-asbestos challenge attenuates pulmonary fibrosis in mice at 60 days. Fibrosis determined on day 60 in mouse lung. Trichrome staining (**A**) and fibrosis score (**B**) of lung specimens. Collagen levels by Sircol assay (**C**). *n* = 4−10; * *p* < 0.05 vs. Ti or Ti+PF543; ^†^ *p* < 0.05 vs. CROC. Scale bars: 100× = 0.1 mm; 400× = 0.05 mm.

We next explored the protective effects of PF543 against asbestos-induced lung fibrosis when administered following asbestos exposure. Mice were exposed to a single IT-instillation of crocidolite asbestos (100 μg /50 μL PBS) or nonfibrogenic control particulate (TiO_2_ 100 μg /50 μL PBS) for either 21 d (short-term) or 60 d (long-term) and following exposure were treated with either PF543 (1mg/kg) or vehicle IP injection every other day from day 14−21 or day 30−60 (Figure 1) [21,35]. Consistent with our prior studies [6,29,31], compared to TiO_2_, asbestos fibers increased pulmonary fibrosis at 21 d in B6 mice as evidenced by histology (H&E and trichrome staining–Figure 4A), lung fibrosis score assessed by a pathologist blinded to the experimental protocol (Figure 4B), and lung collagen levels (Figure 4C). PF543 post-treatment begun 7 d following asbestos IT-instillation significantly reduced asbestos-induced lung fibrosis at 21 d as compared with untreated mice (Figure 4). Notably, asbestos-induced lung fibrosis persisted at 60 d was also reduced by PF543 post-treatment that was begun 30 d following asbestos IT-instillation as assessed by histology, lung fibrosis score, and lung collagen levels (Figure 5). Collectively, these studies firmly support that SPHK1 inhibition with PF543 begun following exposure to two different fibrogenic agents can mitigate pulmonary fibrosis.

### 2.2. PF543 Attenuates Asbestos-Induced S1P Production and mtDNA Damage in Mouse Lungs and MLE Cells

**Figure 6:**
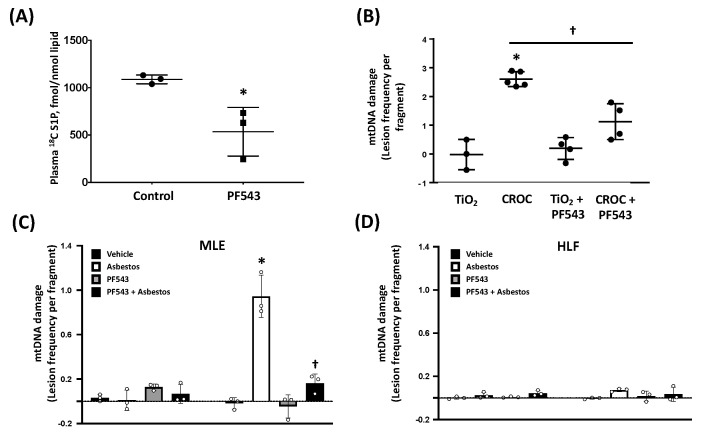
PF543 attenuates plasma S1P, lung mtDNA damage in asbestos-exposed mice at 21 d, and asbestos-induced mtDNA damage in Alveolar epithelial cell line MLE 12 (AEC-MLE-12) cells but not Human lung fibroblasts (HLF). Baseline plasma S1P levels (**A**) mtDNA damage in mouse lungs (**B**), and DNA damage in the nucleus and mitochondria of MLE-12 cells (**C**) and HLF (**D**). *n* = 3−5 * *p* < 0.05 vs. TiO_2_, ^†^ *p* < 0.05 vs. CROC; and HLF (**D**), *n* = 3−5, * *p* < 0.05 vs. TiO_2_, ^†^ *p* < 0.05 vs. CROC.

To investigate possible mechanistic pathways accounting for the protective effects of PF543 in our model, several pathways were explored. Given the direct association between plasma S1P levels and IPF mortality and animal models of lungs [3,4,15,20] and that SPHK1 inhibition mitigates bleomycin-induced increases in plasma SIP levels [15], we assessed whether PF543 ameliorates plasma S1P levels when administered every other day at 1mg/kg [35,36]. As shown in Figure 6A, compared to vehicle-treated controls, PF543 post-exposure reduced plasma S1P levels at 21 d by ~50% (S1P levels [pmol/mL]: vehicle challenge- 1153 ± 60 vs. PF543- 540 ± 240; *p* < 0.05; *n* = 3) demonstrating the efficacy of PF543 in reducing plasma S1P levels consistent with prior studies [35,37].

We recently reported that the protective effects of SPHK1 inhibition are due in part to reductions in human lung (HLF) mitochondrial ROS production that occurs following exposure to either bleomycin or TGFβ [21]. Given that mitochondrial ROS and subsequent mtDNA damage can promote AEC apoptosis and lung fibrosis [1,6,7,8,9,10,22,23,24,25,26,27,28], we assessed whether PF543 reduces asbestos-induced mtDNA damage in lung tissue in vivo and cultured lung epithelial cells and fibroblasts in vitro. As expected, crocidolite asbestos augments murine lung mtDNA damage at 21 d as compared to TiO_2_ (Figure 6B). Interestingly, PF543 post-asbestos exposure attenuated mtDNA damage by ~60% as compared to untreated asbestos-exposed mice (1.13 ± 0.54 v 2.60 ± 0.23 lesion frequency per mtDNA fragment, respectively; *p* < 0.05; *n* = 3−4). Given that mtDNA release from both AECs and fibroblasts have been implicated in the pathobiology of pulmonary fibrosis [32,33], we determined whether PF543 ameliorates mtDNA damage from asbestos-exposed cultured lung epithelial cells (MLE) and HLF. As expected, asbestos induced significant mtDNA damage in MLE cells (Figure 6C); notably, mtDNA damage was near completely abolished in PF543-treated MLE cells (0.94 ± 0.16 vs. 0.16 ± 0.07 lesion frequency per mtDNA fragment, respectively, *n* = 3−5, *p* < 0.05 vs. control). In contrast to lung epithelial cells, we detected negligible levels of mtDNA damage in asbestos-exposed HLF (Figure 6D). Taken together, these data suggest that PF543 post treatment in asbestos-exposed mice reduces mtDNA damage in the lungs and that PF543 ameliorates asbestos-induced lung epithelial cell mtDNA while fibroblasts are less susceptible to mtDNA damage.

### 2.3. PF543 Diminishes Recruitment of Pro-Fibrotic Mo-AMs

**Figure 7:**
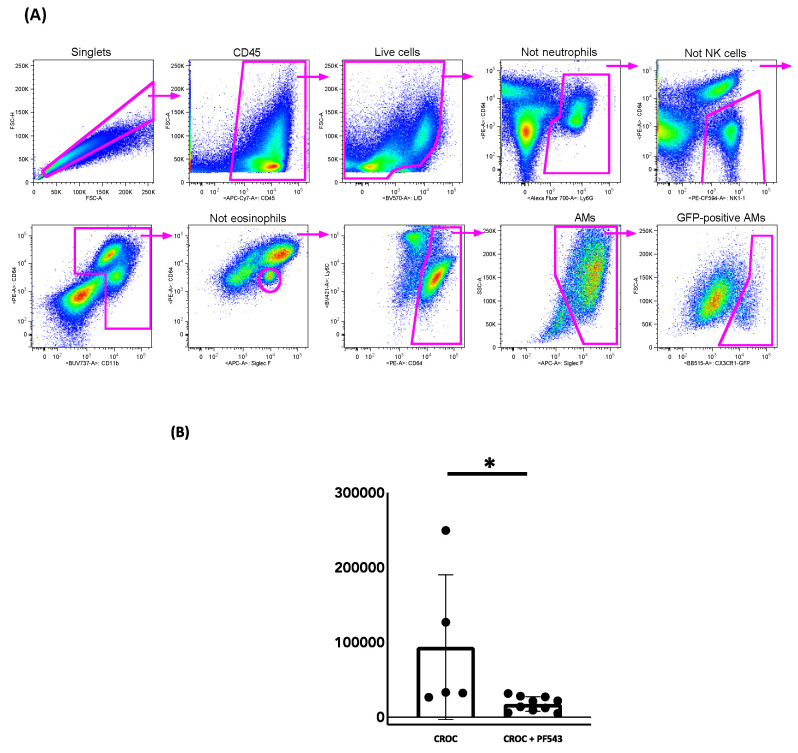
PF543 reduces the number of newly-made “fibrogenic” Mo AMs in mouse lungs exposed to crocidolite asbestos, 60-day post challenge in lungs of CXCR3 ER zsGREEN mice. Flow gating strategy (**A**). Number of new Mo-AMs recruited to lung (**B**). *n* = 4−8, * *p* < 0.05 vs. CROC alone.

We previously showed that genetic ablation of Mo-AMs recruited into the lung in response to the epithelial injury ameliorates bleomycin- and asbestos-induced lung fibrosis and that restoration of the Mo-AM population “rescued” the fibrosis [10,13]. Thus, Mo-AMs have a causal role in the development pulmonary fibrosis. To address whether PF543 impacts the recruitment of Mo-AMs, we used a tamoxifen-inducible fate-mapping system as previously described and validated by our group using the asbestos lung fibrosis model [13,38]. Using this inducible fate-mapping system to assess the kinetics of Mo-AMs recruitment during initiation and maintenance of asbestos-induced pulmonary fibrosis, we reported continuous recruitment of Mo-AMs during the progression of pulmonary fibrosis following asbestos exposure, accounting for 5−8% of all alveolar macrophages at all examined times after exposure along with a gain in the capacity for self-renewal in the fibrotic lung [13]. We leveraged this innovative fate-mapping system to determine whether PF543 post-exposure mitigation of asbestos-induced lung fibrosis at 60 d was due in part to reducing the recruitment of the fibrogenic Mo-AMs. Two weeks before animal harvest, macrophages from zsGreen mice (with the tamoxifen-inducible *Cx3cr1^ER-Cre^* promoter) were green fluorescent protein (GFP) identified by treating animals with 10 mg tamoxifen, followed by a “lineage tracing” flow strategy [13]. Our gating strategy for localizing GFP-positive murine Mo-AMs is shown in Figure 7A. As compared to vehicle-treated asbestos controls, PF543 initiated 30 d following asbestos IT-instillation markedly reduced recruitment of “fibrogenic”, Mo-AMs (93,782 ± 86,419 vs. 17,333 ± 9170 *n* = 5−10, *p* < 0.05 vs. control) (Figure 7B).

## 3. Discussion

Accumulating evidence has firmly established a prominent role for SPHK1/ S1P signaling in the pathobiology of IPF and murine lung fibrosis but the detailed molecular mechanisms involved are not fully understood, nor whether blocking SPHK1 activation following exposure to fibrogenic agents is protective [3,4,15,20,21,39,40]. Herein we address these two important gaps in our understanding of the SPHK1/S1P axis by showing that PF543, a specific SPHK1 inhibitor, administered following exposure to either bleomycin or asbestos, mitigates murine pulmonary fibrosis. We also demonstrate that the beneficial effects of post-exposure PF543 treatment are associated with reductions in lung expression of profibrotic markers, lung mtDNA damage, and the recruitment of fibrogenic Mo-AMs, all of which are important mediators of lung fibrosis.

A major finding in this study is that SPHK1 inhibition initiated following exposure to two different fibrogenic agents significantly reduced pulmonary fibrosis. A strength of our study design involves using two murine models of pulmonary fibrosis for assessing the protective effects of SPHK1 inhibition: (1) bleomycin (spontaneously resolves) and (2) asbestos (nonresolving) [29,34]. Although numerous agents administered before or at the time of bleomycin exposure can mitigate pulmonary fibrosis, the most commonly used murine lung fibrosis model, few have maintained their protective effects when initiated following exposure to bleomycin and we are not aware of any agent showing post-exposure protection in the asbestos model of lung fibrosis [34].

We investigated several mechanisms that may account for the beneficial effects of post-exposure PF543 in ameliorating pulmonary fibrosis. First, we showed that PF543 administered following bleomycin exposure reduces lung expression of markers indicative of ongoing pulmonary fibrosis by activated fibroblasts such as fibronectin, Col1A2, and α-SMA (Figure 3) in a manner that parallels our histologic findings (Figure 2). TGF-β, a key profibrogenic cytokine that is upregulated in the lungs of patients with IPF as well as bleomycin- and asbestos-challenged murine fibrotic lungs [1,3,7,41], can promote lung fibroblast SPHK1 expression and S1P levels as well as trans-differentiation of myoblasts to myofibroblasts via the upregulation of the SPHK1/S1P3 axis [3,15,39,40,42,43,44,45]. Our findings suggest that post-exposure SPHK1 inhibition by PF543 can mitigate these key profibrotic-signaling pathways following bleomycin exposure. Similar to others, we observed that chronic PF543 treatment at 1mg/kg body weight, reduces S1P levels by 50% (Figure 6A) [35,36]. Further, the protective effects noted herein are unlikely caused by off-target effects of PF543 as we and others have previously noted that PF543 doses up to 5 mg/kg body weight in mice for various time points results in no observable cytotoxicity, loss of body weight, or adverse lung or cardiac pathology [35,37,46].

A second mechanistic pathway that we explored was whether SPHK1 inhibition can reduce mtDNA damage in the lungs and AEC, which is crucial for promoting AEC mitochondria-regulated apoptosis and pulmonary fibrosis [1,6,7,8,9,10,22,23,24,25,26,27,28]. Herein, we focused on the murine asbestos lung fibrosis model because, unlike bleomycin, asbestos-induced pulmonary fibrosis does not resolve spontaneously but progresses slowly [34]. We demonstrate that PF543 administered every other day over 21 d reduces plasma S1P levels as expected and that post-exposure PF543 mitigates asbestos-induced lung mtDNA damage (Figure 6A,B). Our in vitro studies show that PF543 ameliorated asbestos-induced AEC mtDNA damage while mtDNA damage in HLF was negligible (Figure 6 C,D). Our findings that PF543 protects asbestos-exposed lung epithelial cells are in accord with a study showing that S1P can promote mitochondria-regulated apoptosis in rhabdosarcoma cells [47] and that SPHK1 deficiency protects mice from acetaminophen-induced endoplasmic reticulum (ER) stress and mitochondrial permeability transition [48].

The mechanism by which PF543 attenuates mtDNA damage is not established from our studies but several lines of evidence suggest it results from reduced mitochondrial ROS levels. Work by our group and others have established that mitochondrial ROS are crucial for promoting mtDNA damage evident in humans with IPF and animal models of pulmonary fibrosis [6,7,8,9,10,11,22,23,24,25,26,27,28,29,30,31,33,49]. Further, we recently showed that inhibition of SPHK1 with PF543 reduced bleomycin- and TGF-β-mediated mtROS levels in HLF and that MitoTEMPO, which scavenges mitochondrial ROS, ameliorated TGF-β-dependent expression of FN and α-SMA, suggesting a direct link between SPHK1-driven mitochondrial ROS levels and fibroblast differentiation. [21]. A role for mitochondrial ROS is also supported by our recent study showing that diminishing AEC mitochondrial ROS production using *MCAT* mice that globally overexpress mitochondria-targeted human catalase reduces mtDNA damage in the setting of oxidative stress and subsequent AEC apoptosis and lung fibrosis following exposure to either asbestos or bleomycin [6].

Although not examined in this study, PF543 may also mitigate mtDNA damage by augmenting mtDNA repair pathways, such as mtOGG1 and/or other BER enzymes. MtOGG1 may be of particular interest given that mtOGG1 prevents oxidant-induced AEC apoptosis by mitigating mtDNA damage [7,8,49] and that asbestos- and bleomycin-induced lung fibrosis is increased in *Ogg1^-/-^* mice but reduced in mice globally overexpressing the human mitochondria-targeted OGG1 subunit 1-alpha transgene (*mtOgg1^tg^*) with concordant changes in AT2 cell mtDNA damage and apoptosis [29,31]. MtDNA release, which was not investigated here, can occur from TGFβ-exposed HLF and apoptotic AECs, the latter of which can promote TLR9 pro-fibrotic signaling [32,33]. Interestingly, there is a direct association between mtDNA levels in the BAL fluid or plasma and mortality in patients with [32]. Taken together, our new findings suggest that SPHK1 inhibition can reduce lung and AEC mtDNA damage that promotes lung fibrosis. Further studies are warranted determining the causal relationship between S1P/SPHK1 signaling and mtDNA release and repair in the pathobiology of lung fibrosis.

A third mechanism that we investigated was whether SPHK1 inhibition by PF543 can prevent the recruitment of profibrotic Mo-AMs. Work from our group and others have established a causal role for Mo-AMs in the pathobiology of pulmonary fibrosis in mice exposed to bleomycin or asbestos as well as in humans with IPF [12,13,14,50,51]. In this study, we used a novel monocyte fate-mapping system for showing that post-treatment PF543 reduces recruitment of the fibrogenic Mo-AMs at 60 d following exposure to asbestos (Figure 7B). Using single-cell RNA sequencing in the murine asbestos lung fibrosis model, we recently established that spatially restricted multicellular pro-fibrotic niches occur during the development of lung fibrosis that includes injured epithelial cells, recruitment of Mo-AMs that are capable of self-renewal, and fibroblast activation and proliferation [13]. PF543 may impact SPHK1 signaling in all these cell types involved in the pathobiology of pulmonary fibrosis since SPHK1 is present in all mammalian cells and generates S1P that can act via its receptors on different types of target cells through diverse mechanisms involving vascular permeability, inflammatory cell infiltration, fibroblast migration, and fibroblast to myofibroblast differentiation. Using cell-targeted conditional SPHK1 knockout mice, we recently reported the specific role of SPHK1 in AECs and fibroblasts, but not endothelial cells, in the development of bleomycin-induced lung fibrosis [21]. Although beyond the scope of this study, it will be of interest determining whether conditional SPHK1 knockdown in macrophages is sufficient for mitigating pulmonary fibrosis.

Our study has some limitations. First, although we have identified several mechanisms that may account for the protective effects of PF543, the detailed molecular mechanisms by which PF543 mitigates mtDNA damage and the recruitment of Mo-AMs warrant further investigation as highlighted above. Second, while we used a validated lineage tracing system to implicate Mo-AMs rather than tissue-resident peribronchial or interstitial macrophages as the source of the protective effects of PF543 in limiting recruitment of pro-fibrotic AMs following asbestos exposure, it is feasible that these other macrophages are important in mediating the beneficial effects of PF543 by directing monocyte recruitment and development [52].

In summary, we found that PF543 administered following exposure to two distinct fibrogenic agents (bleomycin and asbestos) can substantially mitigate lung fibrosis. Further, we show that the protective effects of PF543 occur in part by ameliorating lung expression of profibrotic markers, lung mtDNA damage, and Mo-AM recruitment. Additionally, an important role for PF543 in reducing lung epithelial cell mtDNA damage was suggested by our finding that, unlike HLF that were resistant to asbestos-induced mtDNA damage, we detected substantial lung epithelial cell mtDNA damage following asbestos exposure and PF543 was protective. Given the important role of AEC mtDNA damage in promoting mitochondria-regulated apoptosis and subsequent pulmonary fibrosis, we reason that strategies aimed at limiting activation of the SPHK1 signaling axis is an innovative therapeutic target for managing patients with IPF and other forms of lung fibrosis.

## 4. Materials and Methods

### 4.1. Reagents

Bleomycin and normal saline were purchased from APP Pharmaceuticals (Schaumberg, IL, USA). The Crocidolite asbestos fibers used in this study are Union International Contre le Cancer (UICC) reference standards (a kind gift of Dr. Andy Ghio, U.S. Environmental Protection Agency) [30]. Tamoxifen and purified corn oil were purchased from Millipore-Sigma (St. Louis, MO, USA). Antibodies for Western blotting and Flow cytometry were purchased from commercial vendors and verified previously: see Supplementary Table S1.

### 4.2. Animals

All animal studies described here are approved by the Northwestern University, Jesse Brown VA Medical Center, and University of Illinois at Chicago Institutional Animal Care and Use Committees (IACUCs; NU IACUC protocol, # IS0007912, approved on 29 March 2019 and UIC ACC # 18−201, approved on 20 November 2018). Male and female 8- to 10-week old *C57Bl/6J* wild-type (WT; Jax 00664) and *Cx3cr1ER-Cre×ZsGreen* mice (*zsGreen;* Jax 007906) were used as we previously described [13].

### 4.3. Bleomycin Instillation into Mice

Intratracheal (IT) instillation of bleomycin, or saline to induce lung fibrosis was performed as previously described [6,13,15]. Eight- to ten-week-old male or female WT (*C57Bl/6J*) or zsGreen (*Cx3cr1ER-Cre×ZsGreen)* mice were anesthetized with 3% isofluorane (Butler Animal Health, Dublin, OH, USA), intubated orally with a 20-gauge angiocatheter (BD Biosciences, Sandy, UT, USA) with stock solutions of bleomycin (1.5 mg/kg in 50 µL normal saline, APP Pharmaceuticals), or 50 µL normal saline (control), was instilled in 2 equal aliquots given 2 min apart. After each aliquot, the mice were placed in the right and left decubitus position for 10−15 s.

### 4.4. Asbestos Preparation and Instillation into Mice

IT-instillation of asbestos or TiO_2_ to induce lung fibrosis was performed as described [6,29,31]. Crocidolite asbestos was suspended in Phosphate Buffered Saline (PBS) and 15 mM HEPES at 2 mg/mL and sonicated for 8 min at 40% power (Sonicator: Branson, Danbury, CT, USA) to disrupt fiber clumps. One hundred µg of crocidolite asbestos, or TiO_2_ (negative control particle; Millipore-Sigma, St. Louis, MO, USA), suspended in 50 µL PBS was instilled into mice in 2, 25 µL aliquots.

### 4.5. Lung Harvest and Histology

Lungs were harvested 21 or 60 days after IT instillation of bleomycin, asbestos, or TiO_2_ or saline controls to assess histology as previously described [6,29,31]. Briefly, a 20-gauge cannula was sutured into the trachea, and the right lung was ligated at the hilum after removal of the left lung, which was saved for biochemical collagen determination, was inflated to 15 cm H_2_O with 10% formalin. The right lung was then dehydrated, embedded in paraffin, and serial 5 µm sections were stained for hematoxylin and eosin (H & E) and Masson’s Trichrome.

### 4.6. Lung Collagen Detection

For soluble collagen determination, the left lung was homogenized in acetic using a polytron (Kinematica, Bohemia, NY, USA) followed by dounce homogenization and clearing by centrifugation. Equal volumes of cleared homogenate were subject to the Sircol assay for soluble collagen based on a modified Pico Sirius Red (Millipore-Sigma, St Louis, USA) collagen precipitation assay as previously described by our group, wherein we showed parallels protein expression of lung type I collagen levels [29].

### 4.7. Fibrosis Scoring System

Lung sections were assessed for fibrosis score as previously described by a pulmonary pathologist (AY), blinded to our experimental protocol, as previously described [6,29,31]. Lungs were scored on severity of fibrosis from 0 (no fibrosis) to 4 (severe fibrosis) and also on the extent of involvement which was quantified on a scale of 1 (occasional alveolar duct and bronchiole involvement) to 3 (more than half of the alveolar ducts and respiratory bronchioles involved. Severity (0−4) and extent (1−3) were multiplied together to yield the fibrosis score.

### 4.8. Bronchoalveolar Lavage (BAL) Cell/Protein Isolation

The BAL studies were performed as previously described [21] briefly, ice-cold PBS (1.0 mL) was injected to the trachea, the BAL cells were collected by centrifugation, counted, lysed for ELISA assay and Western blotting.

### 4.9. PF543 Treatment of Mice

Wild-type and zsGreen mice were intraperitoneally injected with either PF543 (Cayman Chemical, Ann Arbor, MI, USA); 1 mg/kg dissolved in 5% DMSO/PBS) or vehicle controls every other day, from day 7−14 for 21-day short term experiments, and from day 30−60 for 60-day long-term experiments [21,35,36,37,46].

### 4.10. S1P Determination by Mass Spectrometry

S1P levels from mouse plasma were determined as previously described [15,53]. Briefly, lipids were extracted from plasma by a modified Bligh and Dwyer procedure, using 0.1 N HCl for phase separation. Forty pmol of ^17^C -S1P was used as an internal standard and added during the initial lipid extraction step. S1P content was determined by liquid chromatography/ tandem mass spectroscopy with electrospray ionization using an API 5500 AQTRAP mass spectrometer equipped with a turbo -V ion source and normalized to total sample phospholipid content as previously described [15,54].

### 4.11. Quantitative mtDNA Damage Assay via PCR

We assessed nuclear and mtDNA damage as described previously [8,12,31]. Genomic DNA from cultured cells or paraffin-embedded lungs were extracted using the Qiagen Genomic Tip 20/G and Qiagen DNA Buffer set (Qiagen, Gaithersburg, MD, USA) according to the manufacturer’s protocol. Ex-Taq (Takara, Mountain View, CA, USA) was used for PCR, with specific primers to amplify a mitochondrial genomic in both short and long-form nuclear DNA (beta-globin) [8,12]. For DNA quantification, we used PicoGreen (Thermo-Fisher/Invitrogen, Waltham, MA, USA) using the FL600 microplate fluorescence reader, with excitation and emission wavelengths of 485 and 530 nm, respectively. Mitochondria small fragment data were used to normalize fluorescence from the mitochondria long fragment. The number of mitochondrial lesions was calculated by the equation:

*D* = (1 – 2^−(Δlong − Δshort)^) × 10,000 (bp)/size of the long fragment (bp) (1)

### 4.12. Western Blotting

Immunoblot analysis was performed as we have described previously [8,29,31]. Cells were collected and lysed in protein lysis buffer (Cell Signaling, Danvers, MA, USA) with protein and phosphatase inhibitors (Thermo-Fisher/Pierce, Rockford, IL, USA) and cleared by centrifugation. Protein concentration of the supernatant was quantified, boiled in laemmli sample buffer for 5 min. Cell lysate (20ug) was separated on gradient or 10% PAGE gels, transferred to nitrocellulose, and blocked with 5% BSA before overnight incubation with primary antibodies, diluted 1/1000 at 4 degrees C, followed by HRP conjugated secondary antibody incubation, diluted 1/2000, for 1 h at room temperature (Supplementary Table S1). An ECL chemiluminescence kit (GE Healthcare/Sigma, St. Louis, MO, USA) was used for visualization on X-ray film, and bands were quantified (as integrated density) using ImageJ software (NIH, Bethesda, MD, USA).

### 4.13. Cell Culture

The murine lung epithelial-12 (MLE-12) cell line was purchased from the American Type Culture Collection (ATCC, Manassas, VA, USA) and maintained in DMEM (Invitrogen, Grand Island, NY, USA) with 2mM *L*-glutamine supplemented with 10% fetal bovine serum, penicillin (100 units/mL) and streptomycin (100 µg/mL). Cells were plated in 6-well plates or 100-mm dishes and grown to 90% confluence before pre-treatment with PF543 (2 µM in media for 24 h) or PBS/DMSO and/or adding asbestos (25 µg/well) for up to 24 h. Protein extracts were harvested for Western blotting or, in separate experiments, for obtaining nuclear and mitochondrial DNA for DNA fragmentation and a PCR-based DNA damage assay.

Primary human lung fibroblasts were purchased from Lonza (Walkersville, MD, USA), and grown to 80% confluency in 6-well dishes in DMEM medium containing 10% fetal bovine serum and 1% penicillin/ streptomycin. Cells were serum-starved for 24 h before treatment, with crocidolite asbestos, or PBS control, in the presence/absence of PF543 (2 µM in media), or PBS/DMSO alone for 24 h [55].

### 4.14. Induction of Transgene Expression in zsGreen Mice

To address whether PF543 impacts the recruitment of Mo-AMs in fibrotic foci, we used a tamoxifen-inducible fate-mapping system as described [38] and adapted and validated by our group using the asbestos lung fibrosis model [13]. Briefly, after crossing the *Cx3cr1CreER* mice (Jax 020940) with the zsGreen reporter mice, mice were given 10 mg of tamoxifen via oral gavage had 100% of circulating monocytes, which express high levels of *Cx3cr1*, permanently labeled with bright green fluorescent protein (Cre removes floxed STOP sequence in front of the eGFP construct). These labeled monocytes gradually disappear from the circulation over the course of 5–7 days [13,38]. All cells, derived from these monocytes, also are permanently labeled with GFP. Tissue-resident alveolar macrophages do not express *Cx3cr1* and are not labeled in this system. Similarly, Mo-AMs rapidly down-regulate expression of *Cx3cr1* after migration into the alveolar space. Thus, Mo-AMs recruited into the lungs during the 7-day window after a pulse of tamoxifen can be unambiguously identified using the GFP label [13]. Transgene expression in zsGreen mice was induced by oral gavage of tamoxifen, dissolved in sterile corn oil (Millipore-Sigma, St Louis, MO, USA), 10 mg/100 µL, on two consecutive days, one week before harvest. [13].

### 4.15. Cell Isolation, Staining, Flow for Mo-AMs

Cell isolation from lung tissues, flow staining and analysis were all performed as described previously [10,13]. Briefly, after mouse euthanasia and lung perfusion through the right ventricle with 10 mL HBSS, mouse lungs were treated with collagenase D (2 mg/mL, Millipore-Sigma, St. Louis, MO, USA) and DNAse 1 (Millipore-Sigma, St Louis, MO, USA), dissolved in HBSS, with Ca^2+^ and Mg^2+^, using a 30 GA syringe. Lungs were chopped with scissors, transferred to a C-Tube (Miltenyi, Auburn, CA, USA), and processed in a GentleMACS dissociator (Miltenyi) to generate a single-cell suspension, which was then filtered through a 40-um nylon cell strainer. CD 45+ cells were subject to positive selection: the cell suspension was incubated with CD 45+ microbeads, then collected into a MultiMACS Cell 24 separator (Miltenyi). After staining with an AOPI (acridine orange/ propidium iodide, Nexelcom, Lawrence, MA, USA) reagent, cells were counted on a Cellometer K2 automatic cell counter (Nexelcom). After staining with fixable viability dye (eFluor 506, eBioscience, San Diego, CA, USA) and FcBlock (BD, Biosciences, San Jose, CA, USA), cells were stained with the following antibody panel: MHCII:2G9 (BUV395, BD), Ly6C:HK1.4 (eFluor450, eBioscience), CD45: 30-F11 (FITC-fluorescein isothiocyanate, eBioscience), CD64 X54-5/7.1 (PE-phycoerythrin, BioLegend, San Diego, CA, USA), Siglec F: E50-2440 (PECF594, BioLegend), CD11c: HL3 (PECy7,BD), CD24: 1/69 (APC-allophycocyanin, eBioscience), CD11b: M1/70 (APC Cy7, BioLegend), Ly6G:1A8 (Alexa 700, BD), NK1.1: PK (Alexa 700, BD). BD CompBeads and Arc beads (Thermo-Fisher/Invitrogen, Carlsbad, CA, USA) were used for preparation of single-color controls. Flow cytometry and data acquisition were performed at the Northwestern University Robert H Lurie Comprehensive Cancer Center Flow Cytometry Core Facility (Chicago, IL, USA), using a custom-designed BD FACS Symphony instrument using BD FACS Diva software (BD, San Jose, CA, USA). We used FlowJo software (TreeStar, Ashland, OR, USA www.flojo.com) for compensation and data analysis. As described in detail elsewhere [10,13], we used a sequential gating strategy (Supplementary Figure S1) and obtained the cell count for each gate by multiplying the live cell percentages from the Cellometer automatic cell counts by the percentage of cells in the live/singlets gate.

### 4.16. Statistical Analysis

The results of each experimental in vitro condition were determined from the mean of duplicate or triplicate trials. The data were expressed as the means ± SD (*n* = 3 unless otherwise stated). For the in vivo studies, 6 animals per group were used unless noted. An independent sample two-tailed Student’s *t*-test was used to assess the significance between two groups. Analysis of variance was used when comparing more than two groups to a single control; differences between two groups within the set were analyzed by a Fisher’s protected least significant differences test. Probability values < 0.05 were considered significant.

## Supplementary Materials

Supplementary materials can be found at https://www.mdpi.com/1422-0067/21/16/5595/s1. Figure S1. Complete gating strategy; Figure S2. Uncropped blots for Figure 3; Table S1. Antibodies and Key Reagents Used.

## Abbreviations

ATF3	Activating transcription factor 3
**α**-SMA	alpha-smooth muscle actin
AEC	alveolar epithelial cell
AT2	alveolar type II cell
BER	base excision repair
Col1A2	collagen-1A2
ER	endoplasmic reticulum
H&E	hematoxylin and eosin
HLF	human lung fibroblasts
*mtOgg1^tg^*	human mitochondria-targeted OGG1 subunit 1-alpha transgene
IPF	idiopathic pulmonary fibrosis
IP	intra-peritoneal
IT	intra-tracheal
MCAT	mitochondrial catalase enforced-expression
mtDNA	Mitochondrial DNA
mtOGG1	mitochondrial 8-oxoguanine-DNA glycosylase 1
OGG1	8-oxyguanine DNA glycosylase
PINK1	PTEN-induced putative kinase 1
ROS	Reactive oxygen species
SPHK1	sphingosine-kinase 1
S1P	sphingosine-1-phosphate
S1PL	S1P lyase
TGFβ	transforming growth factor-beta
