# Supplementary material for: The Sphingosine Kinase 1 Inhibitor, PF543, Mitigates Pulmonary Fibrosis by Reducing Lung Epithelial Cell mtDNA Damage and Recruitment of Fibrogenic Monocytes
Source: Int J Mol Sci. 2020 Aug 5;21(16):5595. doi: 10.3390/ijms21165595 (PMC7460639; doi:10.3390/ijms21165595)
Supplement: Supplementary file 1 [file ijms-21-05595-s001.pdf]

Supplementary Materials:

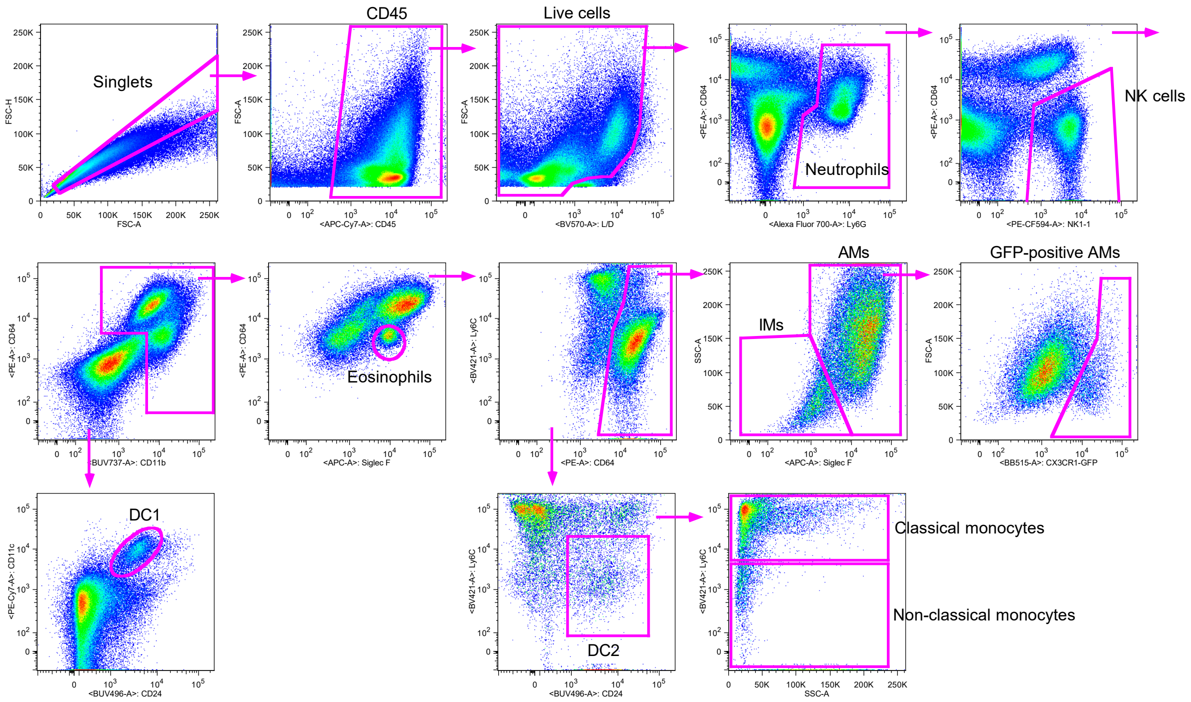

Figure S1. Complete gating strategy.

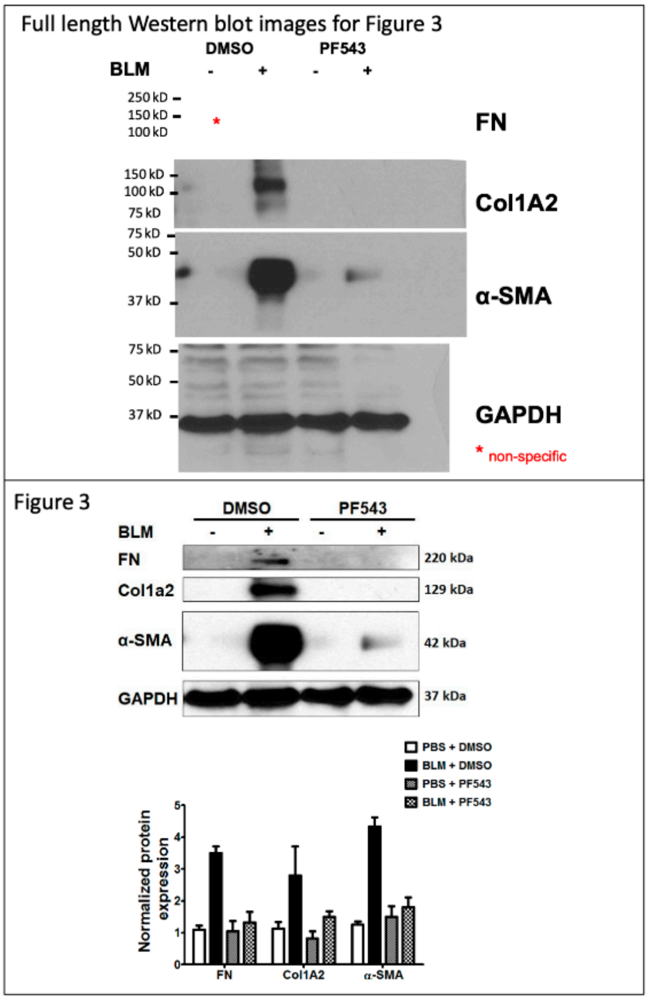

Figure S2. Uncropped blots for Figure 3.

Table S1. Antibodies and Key Reagents Used.

Antibody Name: Clone/KeyReagent	Fluorochrome	MFR
Tamoxifen		Millipore Sigma, St Louis, USA
Corn Oil		Millipore Sigma, St Louis, USA
C57Bl/6J mice		Jackson Labs #00664, Bar Harbor, ME, USA
zsGreen mice		Jackson Labs #007906, Bar Harbor, ME, USA
Bleomycin		APP Pharmaceuticals, Schaumberg, IL, USA
Normal Saline		APP Pharmaceuticals, Schaumberg, IL USA
UICC Crocidolite asbestos		US EPA, Durham, NC, USA
TiO_2		Millipore Sigma, St Louis, MO, USA
Picosirius Red		Millipore Sigma, St Louis, MO, USA
PF543 (SPHK1 inhibitor)		Cayman Chemical, Ann Arbor, MI, USA
Qiagen Genomic Tip/Buffer set		Qiagen, Gaithersburg, MD, USA
ExTaq		Takara, Mountain View, CA, USA
PicoGreen		Thermo-Fisher/Invitrogen, Waltham, MA, USA
FN (rabbit)		Santa Cruz Biotech, Dallas, TX, USA
Col 1A2 (rabbit)		Santa Cruz Biotech, Dallas, TX, USA
TGF-B (rabbit)		Santa Cruz Biotech, Dallas, TX, USA
a-SMA (mouse)		Sigma Aldrich, St Louis, MO, USA
IgG-HRP conjugated		Bio-Rad, Hercules, CA, USA
Collagenase D		Millipore Sigma, St, Louis, MO, USA
DNAse 1		Millipore Sigma, St, Louis, MO, USA
CD 45 positive selection beads		Miltenyi Biotech, Auburn, CA, USA
Fc Block		BD Biosciences, Sandy, UT, USA
Fixable Live/Dead stain	eFluor 506	eBiosciences, San Diego, CA, USA
MHC II: 2G9	BUV395	BD Biosciences, Sandy, UT, USA
Ly6C: HK1.4	eFluor450	eBioscience, San Diego, CA, USA
CD45: 30-F11	FITC (Fluorescein isothiocyanate)	eBioscience, San Diego, CA, USA
CD64: X54-5/7.1	PE (phycoerythrin)	BioLegend, San Diego, CA, USA
Siglec F: E50-2440	PECF594	BioLegend, San Diego, CA, USA
CD11c: HL3	PECy7	BD Biosciences, Sandy, UT, USA
CD24: 1/69	APC (allophycocyanin)	eBioscience, San Diego, CA, USA
CD11b: M1/70	APC Cy7	BioLegend, San Diego, CA, USA
Ly6G: 1A8	Alexa 700	BD Biosciences, Sandy, UT, USA
NK1.1: PK	Alexa 700	BD Biosciences, Sandy, UT, USA
BD Compensation beads		BD Biosciences, Sandy, UT, USA
Arc Beads		Thermo-Fisher/Invitrogen, Waltham, MA, USA
